# Compound C, a Broad Kinase Inhibitor Alters Metabolic Fingerprinting of Extra Cellular Matrix Detached Cancer Cells

**DOI:** 10.3389/fonc.2021.612778

**Published:** 2021-02-25

**Authors:** Mohammed Razeeth Shait Mohammed, Raed Ahmed Alghamdi, Abdulaziz Musa Alzahrani, Mazin A. Zamzami, Hani Choudhry, Mohammad Imran Khan

**Affiliations:** ^1^ Biochemistry Department, Faculty of Science, King Abdulaziz University, Jeddah, Saudi Arabia; ^2^ Cancer Metabolism and Epigenetic Unit, Biochemistry Department, Faculty of Science, King Abdulaziz University, Jeddah, Saudi Arabia; ^3^ Cancer and Mutagenesis Research Unit, King Fahd Medical Research Centre, King Abdulaziz University, Jeddah, Saudi Arabia

**Keywords:** AMP-activated protein kinase, metabolomic analysis, extra cellular matrix detachment, compound C, oxidative phosphorylation

## Abstract

Most of the cancer related deaths are caused mainly by metastasis. Therefore, it is highly important to unfold the major mechanisms governing metastasis process in cancer. Throughout the metastatic cascade, cells need the ability to survive without attachment to neighboring cells and the original Extra Cellular Matrix (ECM). Recent reports showed that loss of ECM attachment shifts cancer cell metabolism towards glycolysis mostly through hypoxia. However, AMPK, a master metabolic regulator was also found to be upregulated under ECM detached conditions. Therefore, in this work we aimed to understand the consequences of targeting AMPK and other metabolic kinases by a broad kinase inhibitor namely Compound C in ECM detached cancer cells. Results showed that Compound C impacts glycolysis as evident by increased levels of pyruvate, but reduces its conversion to lactate thereby negatively regulating the Warburg effect. Simultaneously, Compound C induces block at multiple levels in TCA cycle as evident from accumulation of various TCA metabolites. Interestingly Compound C significantly reduces glutamine and reduced glutathione levels, suggesting loss of antioxidant potential of ECM detached cancer cells. Further, we found increased in metabolites associated with nucleotide synthesis, one carbon metabolism and PPP pathway during Compound C treatment of ECM detached cells. Finally, we also found induction in metabolites associated with DNA damage in ECM detached cancer cells during Compound C treatment, suggesting DNA damage regulatory role of metabolic kinases. Overall, our results showed that Compound C represses pyruvate to lactate conversion, reduces antioxidant potential and invokes DNA damage in ECM detached cancer cells. Our data provides a comprehensive metabolic map of ECM detached cancer cells that can be targeted with a broad kinase inhibitor, is Compound C. The data can be used for designing new combinational therapies to eradicate ECM detached cancer cells.

## Introduction

Cancer is the deadliest disease worldwide; it cost nearly 9.6 million lives in 2018. Most cancer deaths associated with the metastasis stage occur during cellular detachment from the primary site of cancer tumour and get attached to secondary metastatic site for growth (1, 2). Cell adhesion to the ECM-extracellular matrix that helps in cell survival and proliferation signal. In the absence of ECM or cell to cell adhesion leads cells to undergo programmed cell death (apoptosis), termed as *anoikis* (3). Metastatic ECM detached cells develop resistance to anoikis for its survival and re-attach to the distal secondary site to develop metastasis tumor. The signaling pathway involved in regulating cells during anoikis resistance is little known. PKB- The serine/threonine-protein kinase Akt regulates several cellular processes in tumors, including proliferation and cell metabolism (4). the AMP-activated protein kinase (AMPK) is regulated under metabolic stress conditions to maintain cell homeostasis by switching energy metabolism. AMPK inhibits anabolic pathway to minimize ATP consumption, sterol and lipid biosynthesis, glycogen synthesis, and cell cycle progression. AMPK promotes a catabolic pathway to restore ATP and increase glucose uptake, autophagy, lipid utilization, and mitochondrial biogenesis (5–7). AMPK is regulated positively by activation of Try172 residue phosphorylation by LKB1 and CaMKKβ kinase. Recent studies showed the AMPK pro-tumorigenic activity under glucose deprivation and hypoxic condition (7–10).

Glucose deprivation reduces NADPH/GSH level and increases H_2_O_2_ due to impaired PPP. It increases the non-metabolizable 2deoxyglucose(2DG) analogy of glucose, 2DG inhibits glycolysis by mimic glucose starvation and induce cell death (11). AMPK activation protects cancer cells from chemotherapy-induced apoptosis and metabolic stress. Dorsomorphin, also known as Compound C, has the property of inhibiting AMPK that directly effect on blocking metabolic action of AMPK. Dorsomorphin has been effectively anti-apoptotic action of AMPK and causes programmed cell death in many cancer cells type (12, 13).

In this study, we demonstrated the metabolic alteration of dorsomorphin (Compound C) in ECM detached different cancer cells. Interestingly we found dorsomorphin modulates metabolic fingerprinting in cancer cells.

## Materials and Methods

### Cell Culture

The human cancer cell line HCT116 and 22RV1 were kindly gifted from Dr. Hani (Cancer and the epigenetic unit, King Abdulaziz University, Saudi Arabia). Cells were grown at 37˚C with 5% CO_2_, in a Dulbecco’s Modified Eagle Medium (DMEM) with 10% fetal bovine serum (Sigma).

### Matrix Detachment Model

The matrix detachment was done in cell suspension culture. 1 × 10^6^ cells were cultured in ultra-low attachment plate (14) for at 37°C for various time points, which results in formation of spheroids. The spheroids were treated with either vehicle control or with different concentration of compound-C (sigma- P5499) for 5 days. The images were captured by using Nikon (USA) inverted light microscope. Images were analyzed for size measurement using image J software (https://imagej.net/Invasion_assay).

### Apoptosis Assay

The apoptosis was detected by using Annexin V-FITC labelled and propidium iodide followed by a flow cytometer. The cells were grown in an ultra-low attachment plate for 6 days with and without treatment with Compound C. After treatment spheroids are harvested and washed with PBS (ice cold) three times. The spheroids were breakdown by multiple pipetting and resuspended in 100 μl 1X binding buffer and 10 μl Annexin V-FITC and 5 μl PI. After incubation for 20 min in RT (dark condition). The cells were analyzed by flow The Guava^®^ easyCyte 5 Flow Cytometer, and the percentage of cells went apoptosis was calculated (15).

### Metabolites Extraction

Metabolites were extracted from ECM attached and detached cells and detached cells were treated with dorsomorphin (Compound C, Sigma P5499).ECM detached spheroids were collected and crushed immediately using tissue homogenizer using a combination of ice-cold methanol: acetonitrile: water at a ratio of (2:1:1 v/v) and vortexed for 30s and incubated for 60 min at −20°C, and spin for 15 min at 13,000 rpm at 4°C.

The supernatant was collected, dried in a vacuum concentrator, and reconstituted in 200 µl of acetonitrile in 0.1% formic acid, vortexed for 5 min and centrifuge for 10 min at 13,000 rpm at 4°C. Finally, the samples were taken for LC-MS/MS analysis (15–17).

### Mass Spectrometry

Samples were analyzed in LC-MS/MS LTQ XL™ linear ion trap instrument (ThermoFisher Scientific). MSn settings, full scan mode scanning range from 100 to 1000 m/z. Helium was used as buffer gas and Nitrogen was used as sheath gas for run 40 arbitrary units were set as flow rate. The capillary temperature was set at 270°C and voltage 4.0 V; spray voltage was set at −3.0 kV.

### Data Analysis

The raw data file was processed using open accesses online XCMS online database. Peaks were searched against human metabolites in the Human Metabolome database. Pathway analysis and statics were Metaboanalyst (15–17).

### Real-Time qPCR Analysis for mRNA Expression

Briefly, RNA was extracted from all the cell lines at the end of different experimental conditions by using RNeasy kit (Qiagen), and reverse transcribed a High capacity cDNA Reverse Transcription kit (applied biosystems). cDNA (1–100 ng) was amplified in triplicate using gene specific primers (**Supplementary Table 2**). Threshold cycle (*C_T_*) values obtained from the instrument’s software were used to calculate the fold change of the respective mRNAs. Δ*C_T_* was calculated by subtracting the *C_T_* value of the housekeeping gene from that of the mRNA of interest. ΔΔ*C_T_* for each mRNA was then calculated by subtracting the *C_T_* value of the control from the experimental value. Fold change was calculated by the formula 2^−ΔΔ^ *^CT^*.

### Protein Extraction and Western Blot Analysis

Various cancer cells (HCT116 and 22RV1) were cultured in T_75_ flask (1 × 10^6^/flask). After 24 h cells of cell plating in ultra-low attachment plates cells were treated with Compound C for indicated dose for consecutive 5 days. The fresh treatment was added every 48 h, following completion of treatment, media was aspirated and cells were washed with cold PBS (pH 7.4) and pelleted in 15ml falcon tubes. Ice-cold lysis buffer (RIPA buffer) was added to the pellet, with freshly added protease inhibitor cocktail (Protease Inhibitor Cocktail Set III, Calbiochem, La Jolla, CA). Then cells were passed through needle of the syringe to break up the cell aggregates. The lysate was cleared by centrifugation at 14000 g for 30 min at 4°C and the supernatant was used or immediately stored at −80°C. For western blotting 12% poly acrylamide gels were used to resolve 30μg of protein, transferred on to a nitrocellulose membrane, Equal loading were confirmed with ponceau staining of the membrane. Then probed with appropriate monoclonal primary antibodies and detected by chemiluminescence scanner by LI-COR after incubation with specific secondary antibodies (18). The primary antibodies as follows as follows Phospho-AMPKα (Thr172) (D4D6D), AMPKα (D5A2), LDHA (C4B5)

## Results

### Compound C Effectively Reduces Proliferation of ECM Detached Cancer Cells

**Figure 1:**
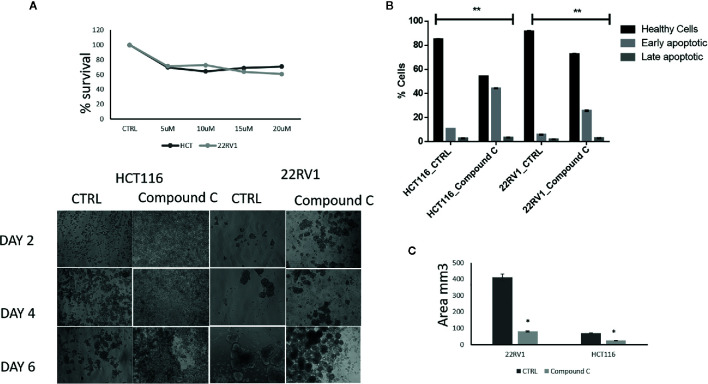
Compound C inhibition reduce cell proliferation and size reduction. **(A)** Cells are grown in ultra-low attachment 96-well plate and treated with different concentration of Compound C and MTT assay were performed. **(B)** Cells are grown in ultra-low attachment plate and treated with Compound C, images were acquired, and size were measured using image J software. **(C)** Cells are grown in ultra-low attachment plat and treated with Compound C; apoptosis assay were performed using Annexin V. * is p-value ≤ 0.05 ** is p-value ≤ 0.01

AMPK is a key regulatory during energy deprivation and activated during ECM detached cancer cells. We examined the toxic effect of Compound C on ECM detached cancer cell proliferation. Results showed that low doses of Compound C i.e. 10 µM in HCT116 and 15µM in 22RV1 Compound C significantly reduced cell proliferation (**Figure 1A**). Prolong treatment of Compound C significantly reduces the spheroid formation and size (**Figure 1B**) and simultaneously induces apoptosis in spheroids formed due to ECM detachment (**Figure 1C**)

### Metabolic Impact of Compound C in ECM Detached Cancer Cells

**Figure 2:**
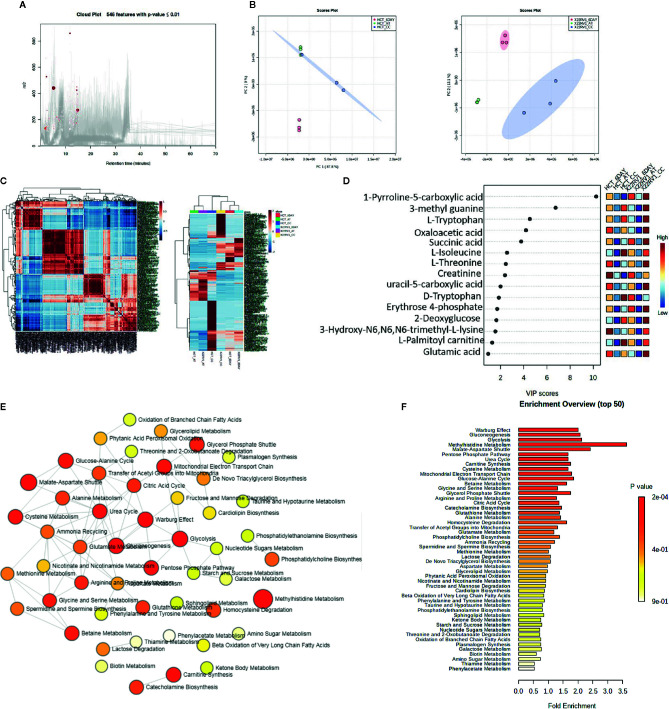
Metabolomic analysis of ECM detached cancer cells and Compound C treated in ECM detached cancer cells, **(A)** Metabolites were extracted and run in LTQ-XL linear ion trap LC-MS and its total ion chromatogram with significate features. **(B)** PCA analysis of total metabolite of HCT 116 and 22RV1 with three different condition. **(C)** Correlation heat map and expression heat map of differential metabolite expressed between ECM attached, ECM detached, and ECM detached cells treated with Compound C. **(D)** VIP score based on PCA analysis of top metabolites. **(E)** Pathway network analysis. **(F)** Top pathway enriched in metabolome analysis between ECM attached, ECM detached, and ECM detached cells treated with Compound C.

To explore the metabolic profile of ECM detached cancer cells treated with broad kinase inhibitor namely Compound C, we performed untargeted metabolomics to comprehend the difference in ECM attached cancer cells with ECM detached cancer cells, and Compound C treated ECM detached cancer cells. Metabolites were acquired using HPLC-MS/MS in DDA- Data Dependent mode, and spectrum raw files were processed using XCMS online database. The total 649 features were identified using ESI positive mode and 546 metabolites (**Figure 2A**) with (p-value ≤ 0.01) statistically significant. Principal component analysis (PCA) models score plots of all samples showed a significant difference in metabolomics between sample groups (**Figure 2B**). The correlation coefficient of the metabolomics data showed metabolites were self-correlations. However, in the Heatmap for the metabolites showed variation in metabolic expression between ECM attached, ECM detached, and ECM detached cells treated with Compound C (**Figure 2C**). The top highly significant metabolite in terms of PCA analysis with expression index showed the variation in metabolite accumulation in Compound C treated ECM detached cancer cells (**Figure 2D**), Top 275 metabolite with expression value shown in **Table 1**. and their raw table with p-value replicates details given in the **Supplementary Table 1**. The pathway enrichment analysis in ECM detached and Compound C treated ECM detached cancer cells compared with ECM attached cancer cells, pathway enrichment, and pathway linkage analysis were obtained through MetaboAnalyst 4.0. A total of 93 metabolic pathways were shown to be enriched (**Figure 2E**), mainly involved in energy metabolisms like Warburg Effect, Gluconeogenesis, Glycolysis, and TCA Cycle. As well as some other metabolic pathways, such as Methyl histidine Metabolism, Pentose Phosphate Pathway, Mitochondrial Electron Transport Chain, urea cycle, and Lactose Degradation, etc. (**Figure 2F**),

**Table 1:** Top metabolite with their expression.

	Forward Primer	Reverse
GLUT1	CATCCCATGGTTCATCGTGGCTGAACT	GAAGTAGGTGAAGATGAAGAACAGAAC
GLUT3-	TGCCTTTGGCACTCTCAACCAG	GCCATAGCTCTTCAGACCCAAG
HK2	GCCATCCTGCAACACTTAGGGCTTGAG	GTGAGGATGTAGCTTGTAGAGGGTCCC
PFKL	GGAGAAGCTGCGCGAGGTTTAC	ATTGTGCCAGCATCTTCAGCATGAG
ALDOA	AGGCCATGCTTGCACTCAGAAGT	AGGGCCCAGGGCTTCAGCAGG
GAPDH	TTCCGTGTCCCCACTGCCAACGT	CAAAGGTGGAGGAGTGGGTGTCGC
PGK1	ATGTCGCTTTCTAACAAGCTGA	GCGGAGGTTCTCCAGCA
PGAM1	GGAAACGTGTACTGATTGCAGCCC	TTCCATGGCTTTGCGCACCGTCT
ENO1	GACTTGGCTGGCAACTCTG	GGTCATCGGGAGACTTGAA
ENO2	TCATGGTGAGTCATCGCTCAGGAG	ATGTCCGGCAAAGCGAGCTTCATC
PKM2	GCCCGTGAGGCAGAGGCTGC	TGGTGAGGACGATTATGGCCC
PDK1	CATGTCACGCTGGGTAATGAGG	CTCAACACGAGGTCTTGGTGCA
PFKFBP1	CTACTGAGCCCTTTCCAAGAA	GCAGAGTAGGAGAAGAGCAAA

### Compound C Reduces Lactate Formation

**Figure 3:**
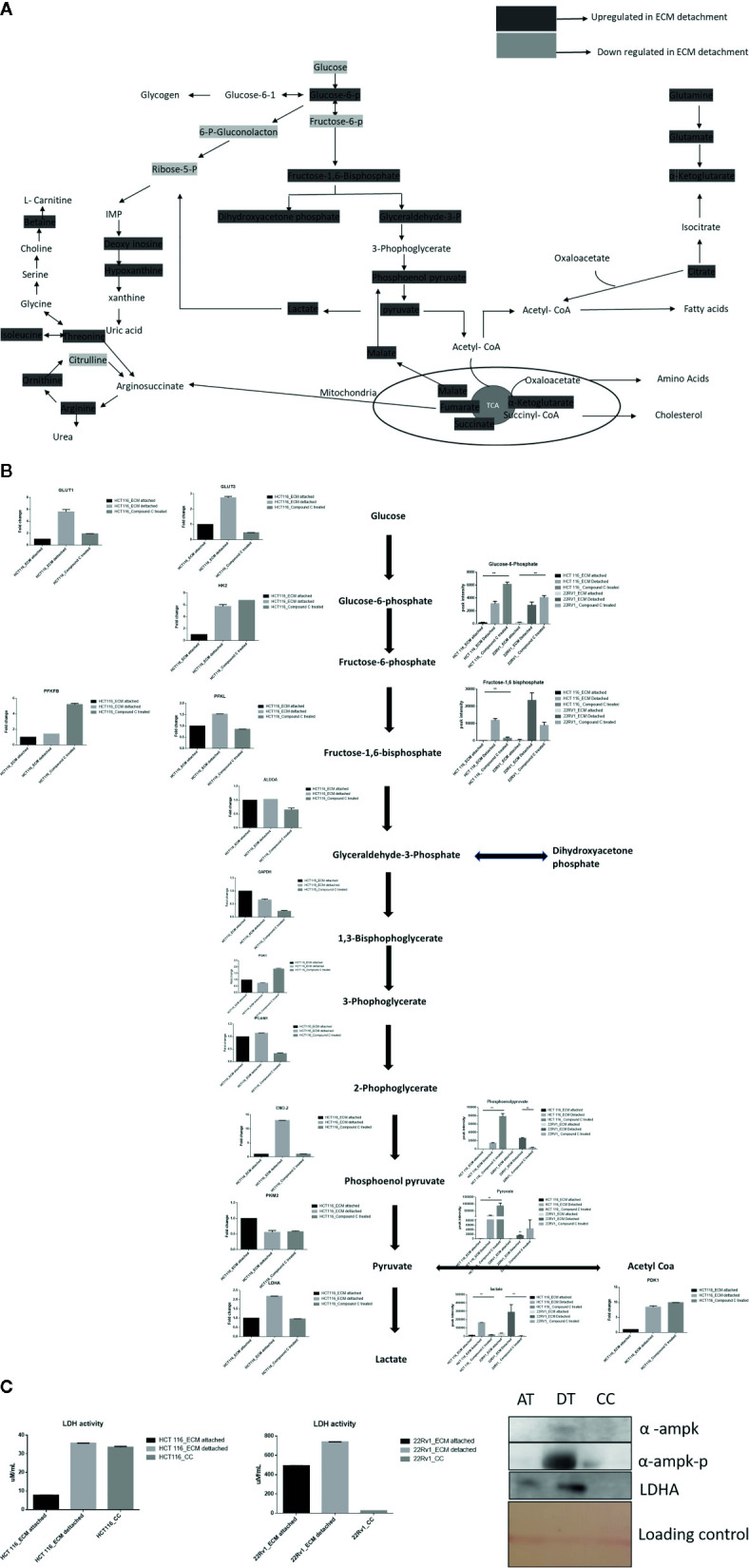
Compound C alter energy enrichment pathway in ECM detachment. **(A)** Overall energy enrichment pathway with metabolite expression index. **(B)** Expression of transcript (HCT116) and metabolite involved in glycolysis during ECM detachment and AMPK inhibited ECM detached cells, P-value p < 0.01. **(C)** LDHA assay and Western blot analysis of protein involved in AMPK activation and glycolysis(AT- ECM attached, DT- ECM detached and CC- Compound C treated).

Lactate production in aerobic glycolysis is a prevalent energy metabolism in cancer cells metabolic pathway. We evaluate mRNA expression and metabolites involved in different steps of glycolysis. Data showed that the mRNA expression of many glycolytic gene namely GLUT1 and 3, Hexokinase 2 (HK2), PFKL (6-phosphofructokinase), PFKFB1 (fructose-2,6-biphosphatase 1), ENO2 (Enolase 2), PGAM1 (Phosphoglycerate Mutase 1), PDK1 (Pyruvate Dehydrogenase Kinase 1), LDHA (Lactate dehydrogenase A), PKM2 (pyruvate kinase M2) and their associated metabolites were high in ECM detached conditions. Compound C reduces both transcripts and associated metabolites of the above-mentioned enzymes in ECM detached cancer cells (**Figures 3A, B** **)**. We further noticed that some enzyme transcript levels i.e. ALDOA (aldolase, fructose-bisphosphate A) GAPDH (glyceraldehyde 3-phosphate dehydrogenase), and PGK1- (Phosphoglycerate Kinase 1) failed to get induced in ECM detached conditions (**Figures 3A, B** **)**.

The reduction of LDHA transcript was well corroborated with lower lactate dehydrogenase activity in Compound C treated cells (**Figure 3C**). These findings suggest that the end product of glycolysis i.e. pyruvate was preferred to enter oxidative phosphorylation rather than lactate formation in ECM detached cancer cells during Compound c treatment. We also observed reduction in phosphorylated-AMPK levels well-known to be targeted by Compound C in treated cells (**Figure 3C**).

### Compound C Treatment Results in Malic Acid Accumulation in ECM Detached Cells

**Figure 4:**
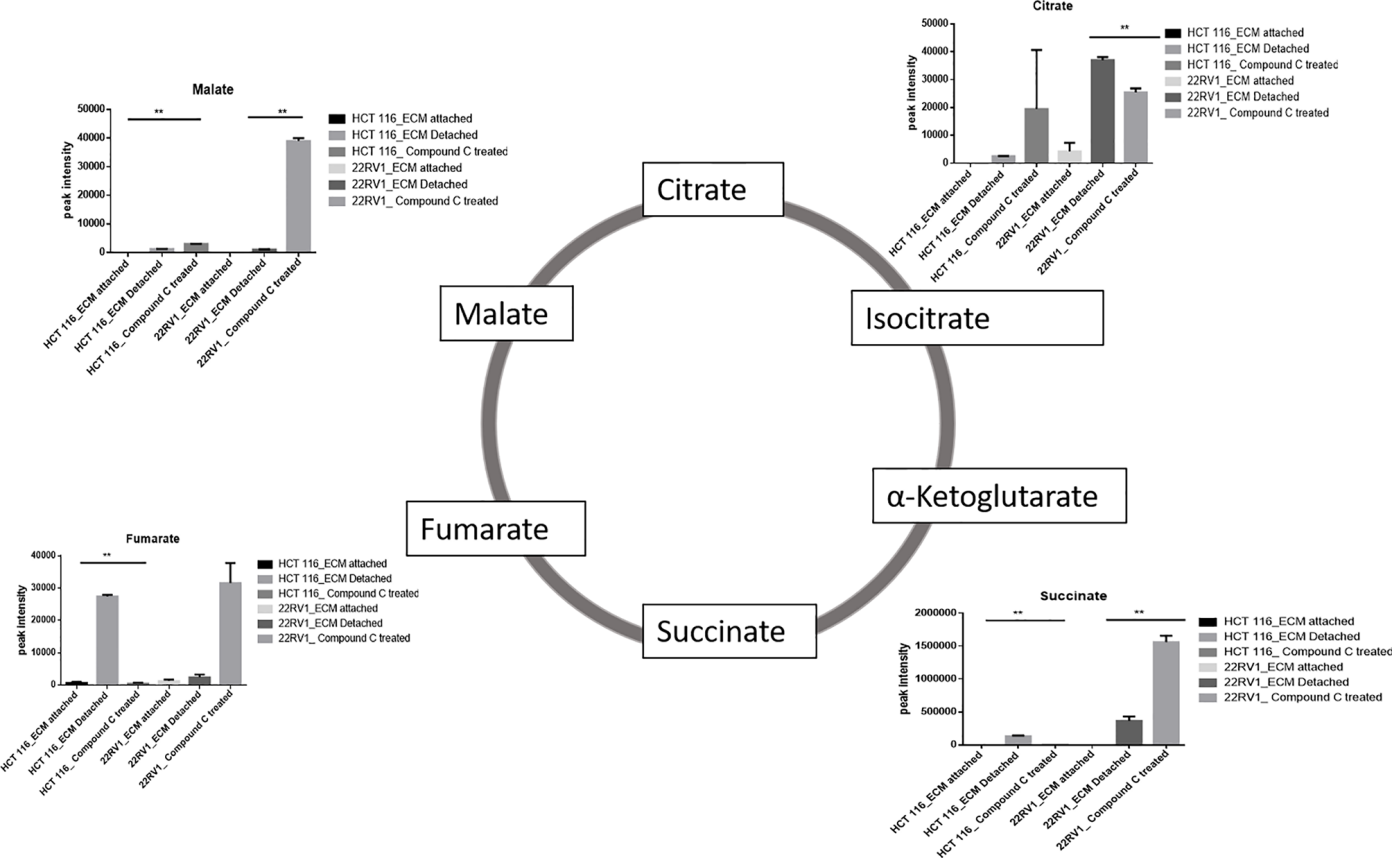
Compound C modulates TCA cycle. ** is p-value ≤ 0.01.

ECM detached cancer cells need metabolic reprogramming to survive and within the known metabolic kinases, AMP-activated protein kinase (AMPK) is a primary regulator of metabolism (19). We found that ECM detached cancer cells have accumulation of various TCA intermediates such as malate and fumarate, suggesting their potential role in ECM detached cancer cell metabolism. Compound C treatment induces block at multiple steps of TCA cycle as evident by accumulation of TCA metabolites. We observed a further increase in both malate and fumarate in both cell lines treated with Compound C (**Figure 4**).

### Compound C Depletes Glutamine and Alters Cellular Antioxidant Levels

**Figure 5:**
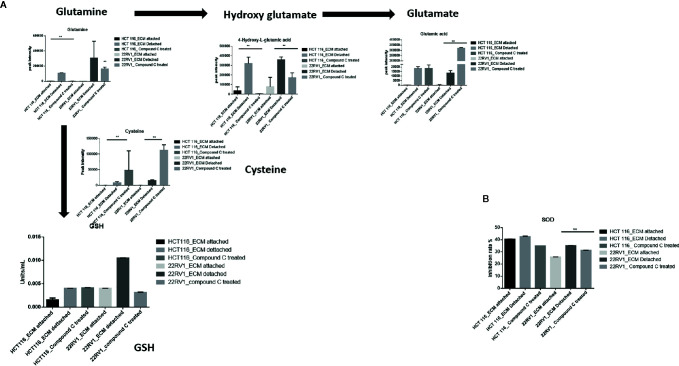
Compound C alters GSH level. **(A)** Overall GSH enrichment pathway with metabolite expression index and GSH assay. **(B)** SOD assay during ECM detachment and AMPK inhibited ECM detached cells, P-value **p < 0.01.

Glutamine have multipurpose roles in cellular metabolism. Glutamine feds TCA cycle along with nucleotide biosynthesis, GSH production and production of nonessential amino acids. We found a significant increase in glutamine and hydroxyglutarate (2HG) in ECM detached cells. 2HG is an oncometabolite. 2HG is known to be produced by mutant IDH ½ enzymes and can also be generated from glutamine derived from αKG (20, 21). Compound C significantly reduces the cellular glutamine levels of ECM detached cancer cells. Since glutamine feeds for reduced glutathione (GSH) production, we measured the GSH levels and as expected ECM detachment showed increased GSH levels which were significantly reduced by Compound C (**Figure 5A**). Next, we measured activity of super oxide dismutase (SOD), reduces ROS, and found that ECM detached cancer cells have high activity and Compound C reduces its activity (**Figure 5B**). Overall, these results showed ECM detached cancer cells possess increase glutamine, GSH and antioxidant capacity and Compound C reduces glutamine, GSH and antioxidant capacity.

### Compound C Favors Ribosugar Synthesis

**Figure 6:**
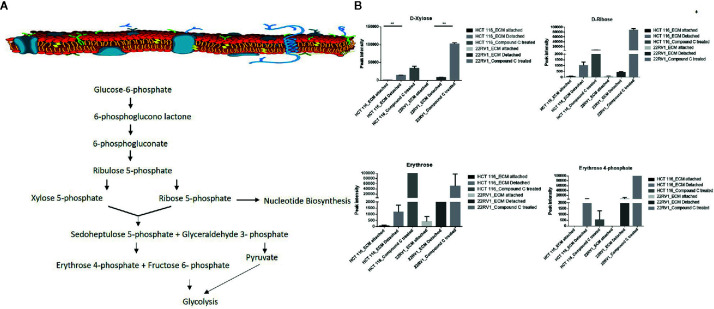
Compound C alter the metabolite involved pentose phosphate pathway. **(A)** Metabolite involved in PPP pathway. **(B)** Expression of metabolite involved in PPP during ECM detachment and AMPK inhibited ECM detached cells, P-value p < 0.01. * is p-value ≤ 0.05 ** is p-value ≤ 0.01

Hexose monophosphate shunt (HMP), also known as Pentose phosphate pathway (PPP) or phosphogluconate pathway, starts from the branch of glycolysis and the first step of glucose metabolism. HMP activation is demonstrated in different types of cancer and in their role in association with metastasis, invasion, and angiogenesis (22, 23). Our studies showed the ECM detached cells increased intermediate metabolite of HMP shunt in both cell lines. Compound C treatment increases ribose, erythrose and xylose levels. However, there were no significant changes observed in nucleotide levels of adenosine and guanosine (**Figure 6**) (**Table 2**).

**Table 2:** Fold change of metabolite involved in PPP- fold change was calculated with normalized value of ECM attached and up and down regulation was mention with proposition to ECM detached and Compound C treated.

	22RV1				HCT116				P value
	ECM Attached	ECM detached	Compound C		ECM Attached	ECM detached	Compound C		
Xylulose 5 phosphate	1	221.4749	70.43583	down	1	45.16325	1387.211	up	0.002851
Sorbitol-6-phosphate	1	35.28718	24.27192	down	1	3.658933	11.68237	up	0.00201
Ribose-5-phosphate	1	0.180434	0.056273	down	1	0.558023	0.930893	up	0.00385
Mannitol 1-phosphate	1	11.58318	8.200933	down	1	0.223793	1.231869	up	0.004667
Guanosine	1	19.38104	15.46899	down	1	1.679476	2.855435	up	0.004705
Guanidinosuccinic acid	1	434.0069	82.62108	down	1	101.89	5994.094	up	0.002381
Erythrose 4-phosphate	1	3434.549	527160.1	up	1	1935.108	366.8125	down	0.019394
Erythrose	1	6.659711	276.3982	up	1	25.87366	18793.61	up	0.002141
D-Xylose	1	115.2781	937.4171	up	1	127.792	257.4589	up	0.003406
D-Ribose	1	8.750765	1606.917	up	1	40.64657	167.1691	up	0.002469
adenosine diphosphate	1	253.3069	138.6028	down	1	7.017809	48.89972	up	0.002551
adenosine	1	134.3279	101.9717	down	1	4.509506	9.377341	up	0.002368

### Compound C Modulates One Carbon Metabolites

**Figure 7:**
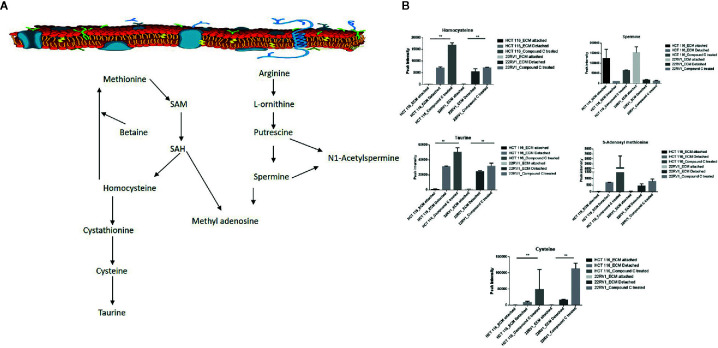
Compound C the modulate metabolite expression of one carbon metabolism **(A)** Metabolite involved in one carbon metabolism pathway. **(B)** Expression of metabolite involved in One carbon metabolite during ECM detachment and AMPK inhibited ECM detached cells, P-value p < 0.01. ** is p-value ≤ 0.01.

One-carbon (1C) metabolism, which sets a broader set of transformation from folate metabolism. 0ne-carbon metabolism is regulatory in cellular physiology. One-carbon metabolism is mostly derived from glycine and non-essential amino acid serin. One- carbon metabolism generate output metabolites that serve as essential building blocks for a redox reaction, biosynthesis, and methylation (24). We sought to verify the functional effect on one-carbon metabolism by Compound C in detached cells; data showed increased one-carbon metabolite intermediates in ECM detached cells such as SAM, L-cystathionine, methyl-cysteine, homocysteine and cysteine suggesting that ECM detached cancer cells might have more methylation phenotype. Coherent with this observation, Compound C treatment in ECM detached cells showed further increase in S-adenosyl-methionine (SAM), homocysteine, and cysteine in both cell lines, but other intermediate metabolites showed different trends between two cell lines (**Figure 7**) (**Table 3**).

**Table 3:** Fold change of metabolite involved in one carbon metabolism- fold change was calculated with normalized value of ECM attached and up and down regulation was mention with proposition to ECM detached and Compound C treated.

	22RV1				HCT116				P value
	ECM Attached	ECM detached	Compound C		ECM Attached	ECM detached	Compound C		
Taurine	1	25.61044	62.29942	up	1	50.94784	95.3329	up	0.001673
Spermine	1	0.119093	0.074492	down	1	0.103608	0.672684	down	0.002103
Selenohomocysteine	1	46.36336	78.14272	up	1	100566.2	2692.212	down	0.002657
S-Adenosyl-l-homocysteine	1	0.398734	0.20135	down	1	0.601573	23.40819	up	0.002926
S-Adenosyl methionine	1	18.2159	20.10414	up	1	43.84777	582.1221	up	0.002815
L-Cystathionine	1	818.562	200.4249	down	1	15.45662	1.346547	down	0.001929
Cytosine	1	106.4612	69.45275	down	1	0.930727	3.676395	up	0.002671
Methylcysteine	1	7.949229	13.74496	up	1	34.12177	99.65528	up	0.003304
Methionine	1	0.999488	0.809505	down	1	11.69524	166.5561	up	0.020447
Homocysteine	1	43.40685	157.0146	up	1	199.278	713.961	up	0.003783
Cysteine	1	110.7534	724.504	up	1	2923.258	112134.5	up	0.003325
Betaine	1	1.888104	0.45807	down	1	2.182797	205.0076	up	0.007133

### Compound C Increase Levels of Metabolites Associated With DNA Damage

**Figure 8:**
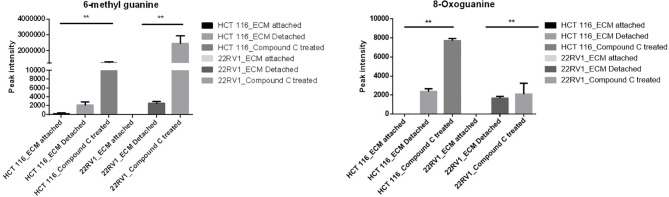
Effect of Compound C on DNA lesion. ** is p-value ≤ 0.01.

Genomic DNA damage is instigated by reactive oxygen species (ROS)generated by aerobic metabolism. Both cytotoxic and mutagenic DNA lesions are associated with oxidative DNA damage (25–27). We noticed increased levels of metabolites namely 8-Oxoguanine, 3’-O-Methylguanosine, 6-methyl guanine and 3-Methylcytosine, associated with DNA damage in ECM detached cancer cells. Compound C further increased their levels suggesting a protective role of AMPK and other metabolic kinases in DNA damage conditions [**Figure 8**, (**Table 4**)].

**Table 4:** Fold change of metabolite involved in DNA lesion - fold change was calculated with normalized value of ECM attached and up and down regulation was mention with proposition to ECM detached and Compound C treated.

	22RV1				HCT116				P value
	ECM Attached	ECM detached	Compound C		ECM Attached	ECM detached	Compound C		
Hypoxanthine	1	4718.953	3303.693	down	1	3206.921	49.92634	down	0.00306
Deoxyinosine	1	46.65531	32.8449	down	1	2295.48	137061.3	up	0.001631
Deoxycytidine	1	517.0447	81.81474	down	1	11740.75	84000.85	up	0.002183
8-Oxoguanine	1	1554.729	25277.78	up	1	2146.321	8091.626	up	0.002033
8-Hydroxyguanine	1	0.10706	0.042731	down	1	0.121629	0.731751	up	0.002844
5-Methylcytosine	1	2.945086	6.408358	up	1	9.156975	0.786644	up	0.003468
3’-O-Methylguanosine	1	44.06217	17.30408	down	1	0.900212	1.214881	down	0.006619
3-Methylxanthine	1	0.25198	0.511269	up	1	5.691069	15.18474	up	0.006005
3-Methylthymine	1	0.013313	0.019806	up	1	0.010739	0.052247	up	0.245542
3-Methylcytosine	1	491678.7	149428.8	up	1	652.269	1197.571	up	0.018722
3-methyl guanine	1	608.754	365505.6	up	1	522.408	6618.538	up	0.002785

## Discussion

Numerous studies have shown that the metabolic reprogramming induced by ECM detachment contributes to cell survival and promotes metastasis. AMPK is a nutrient sensor activated in ECM detachment when ATP : ADP ratio is low (27). In this study, we have shown that metabolic adaption during ECM detachment and during the energy crisis. A metabolic switch happened during Compound C treatment in ECM detached cells. ECM detachment is a signature for metastasis and tumorigenesis (28). Cell depend on glucose metabolism for energy production and biosynthesis need for cell survival and proliferation (29). We showed ECM detachment increase in glycolysis in detached cells. AMPKα1, keeps the high level of reduced glutathione to maintain reduction–oxidation reaction (redox) homeostasis, AMPKα1 regulate the glutathione reductase (GSR) phosphorylation possibly through residue Thr507 which enhances its activity of enzyme (30). Our studies have shown that glutamine levels in ECM detached cells increased and supports metabolic switch reductive carboxylation for energy requirements. Reductive carboxylation is known for mitochondrial disfunction and supports cell proliferation. Our results demonstrated an Compound C was able to reduce glutamine, and other intermediate metabolites. ECM detached activates reductive carboxylation and oxidation glutamine with glycolysis, enabling biomass generation and ATP production; and maintained redox homeostasis, AMPK inhibition reduces glutamine and reduces biomass production and energy deprivation.

ECM detached cancer cells that allow glycolytic intermediate to accumulate and switch to alternate pathway HMP or PPP, which can stimulate macromolecular synthesis and combat oxidative stress (31, 32), our studies showed the ECM detachment activates PPP, intermediate metabolite accumulation was increased on ECM detached cells. The PPP is composed of two functionally interrelated branches: the oxidative and the non-oxidative metabolism. In the oxidative arm of the HMP, three major irreversible reactions involved in the reduction of glucose-6-phosphate to ribose-5-phosphate with reduction of nicotinamide adenine dinucleotide phosphate (NADPH) and ribulose-5-phosphate (Ru5P), In the synthesis of nucleotide (33–35). The non-oxidative branch of the HMP generates nucleotide synthesis from glyceraldehyde-3-phosphate and fructose-6-phosphate. Our studies show the glyceraldehyde-3-phosphate increase in 22RV1 and decrease in HCT116, in both cell lines, AMPK inhibition shows an increase in ribose sugar and erythrose involve in the salvage pathway. In this scenario, we observed that the22RV1 might involve non-oxidative and HCT116 oxidative PPP pathways during AMPK inhibition.

Several pathways are intermediate and generate one-carbon metabolism is include serine and glycine metabolism. Cancer cells show alter DNA, RNA, and histone methylation patterns. Epigenetic modification in DNA, RNA, and histone regulates various gene regulation and function. Post-translational modification (PTM) in protein methylation alter protein-protein interaction (36). Our observation showed, ECM detachment responsible for the activation of epigenetic modulator metabolite involved in one-carbon metabolism. S-adenosylmethionine (SAM) is a methyl donor; SAM transfer the methyl group to DNA and RNA, it converted to S-adenosylhomocysteine, which is converted to homocysteine and recycled to methionine. Importantly, SAM and homocysteine levels increased in ECM detached cancer cells. This observation is showing that methylation might be high in ECM detached cancer cells. The recent studies revealed that cysteine regulates the mTOR1 activity, cysteine uptake inhibits the mTOR activation and halts protein synthesis, and mTOR is regulated by AMPK. Our studies have shown the cysteine level in ECM detached and AMPK inhibited ECM detached cancer cells are increased, it is showing Compound C induces the cysteine level in ECM detached cells.

DNA lesion and DNA damage is ingested by effect of ROS production by cellular level, 8-oxoguanine is from oxidative DNA damage, effect of oxidative DNA damage results in pathophysiological changes (37). 6-methyl guanine is example for in what way DNA lesion leads to development of DNA damage. DNA damage may responsible apoptotic effect and mutagenesis properties, insertion of mutated 6-methyl guanine pass through cell cycle and causes the secondary DNA lesions and infer in DNA replication. 6-methyl guanine break the double standard DNA and triggered apoptosis (37). The data reported here reveal ECM detachment enhanced the expression of antioxidant enzyme GSH and SOD levels and inhibition of AMPK reduce antioxidant enzyme. Reduction in antioxidant enzyme activity may be increase in ROS levels and causes the DNA damage.

The significant consequence of data presented here represent advance in our understanding of ECM detached cancer cells switch the metabolic pathways., promotes cancer cell survival during metastasis. Compound C reduces pyruvate to lactate conversion, promotes OXPHOS, reduces glutamines and reduced GSH levels, and increases metabolite levels associated with DNA damage suggesting a broad metabolic impact. Beside Compound C, some other AMPK inhibitors are available like Doxorubicin, GSK6906893 and metformin. Our data also provides a detailed metabolic map of ECM detached cells, can be used to extrapolate and design future studies to identify novel targets to eradicate ECM detached cancer cells.

## Data Availability Statement

The original contributions presented in the study are included in the article/**Supplementary Material**. Further inquiries can be directed to the corresponding author.

## Author Contributions

MRSM and RA performed the experiment and wrote the first draft of the manuscript. AA performed a flow cytometry experiment. RA and MZ guidance and proofreading of the manuscript. MRSM and HC supervised and reviewed the manuscript. MK proposed, designed, and supervised the entire study and also wrote and proofread the manuscript. All authors contributed to the article and approved the submitted version.

## Funding

This project was funded by the Deanship of Scientific Research (DSR) at King Abdulaziz University (KAU), Jeddah, under grant no. (KEP-15-130-41). The authors, therefore, acknowledge with thanks DSR technical and financial support.

## Conflict of Interest

The authors declare that the research was conducted in the absence of any commercial or financial relationships that could be construed as a potential conflict of interest.

## Supplementary Material

Supplementary Table 1 raw metabolite list from XCMS.

Supplementary Table 2 Primer list.
